# Medial cortical bone thickness of the tibial diaphysis in osteoarthritis is related to lower extremity alignment and tibial morphology

**DOI:** 10.1186/s13018-024-04849-y

**Published:** 2024-06-15

**Authors:** Keisuke Maeda, Tomoharu Mochizuki, Osamu Tanifuji, Ryota Katsumi, Koichi Kobayashi, Hiroyuki Kawashima

**Affiliations:** 1 Division of Orthopedic Surgery, Niigata University Medical and Dental Hospital, 1-757 Asahimachi-dori Chuo-ku, Niigata City, Niigata, 951-8510 Japan; 2 School of Health Sciences, Faculty of Medicine, Niigata University, Niigata, Japan

**Keywords:** Knee osteoarthritis, Healthy, Cortical bone thickness, Tibia, Alignment, Inclination of the medial compartment of the proximal tibia

## Abstract

**Background:**

The purpose of this study was to clarify (1) the differences in cortical bone thickness (CBT) of the tibial diaphysis between healthy and osteoarthritic knees and (2) the influences of the femorotibial angle (FTA) and inclination of the medial compartment of the proximal tibia (MCT) on tibial CBT.

**Methods:**

The study assessed 60 subjects with varus knee osteoarthritis (OA) (22 males and 38 females; mean age, 74 ± 7 years) and 53 healthy elderly subjects (28 males and 25 females; mean age, 70 ± 6 years). Three-dimensional estimated CBT of the tibial diaphysis was automatically calculated for 2752–11,296 points using high-resolution measurements from CT. The standardized CBT was assessed in 24 regions by combining six heights and four areas. Additionally, the association between the CBT, each FTA, and MCT inclination was investigated.

**Results:**

The OA group showed a thicker CBT in the medial areas than in the lateral areas of the proximal tibia, while the healthy group had a thicker lateral CBT. The medial-to-lateral ratio of the proximal tibia was significantly higher in the OA group than in the healthy group. The proximal-medial CBT correlated with FTA and MCT inclinations in the OA group.

**Conclusions:**

This study demonstrated that varus osteoarthritic knees showed a different trend of proximal-medial CBT with associations in FTA and MCT inclination from healthy knees, possibly due to medial load concentration.

## Background

Cortical bone thickness (CBT) varies with the mechanical loads applied to the bone. It is an optimal parameter for assessing structural adaptation due to biological factors and mechanical use. The CBT of the tibia is assumed to change under the influence of the loading environment, including aging, bone mineral density, bone morphology, and whole lower extremity alignment.

Previous studies using the CBT to examine the pathomechanism of knee OA measured using 2D X-rays [1], but in recent years, a highly accurate method that can precisely estimate 3D-CBT from clinical low-resolution CT data has been reported [2–5].

A detailed study of the relationship between 3D-CBT and each of lower extremity alignment and bone morphology by 3D evaluation that our group have previously reported [6–13] will lead to a better understanding of the pathomechanism of knee osteoarthritis (OA).

The purpose of this study was to clarify (1) the differences in the CBT of the tibial diaphysis between healthy and osteoarthritic knees; and (2) the influence of whole lower extremity alignment and inclination of the medial compartment of the proximal tibia (MCT) on tibial CBT.

The hypotheses were as follows: (1) the medial CBT in varus knee OA shows a different trend from healthy knees; and (2) the medial CBT of the proximal tibia correlates with standing lower extremity alignment and MCT inclination.

## Methods

This study was performed in accordance with a protocol approved by the Institutional Review Board of Niigata University (IRB number 2015–2351). All participants provided written or verbal informed consent for participation in the study and for the use of their data.

### Subjects

The inclusion criteria were healthy elderly individuals (age > 50 years) without obesity (body mass index [BMI] < 30] and varus knee OA. The exclusion criteria were as follows: no history of trauma, valgus knee OA, postoperative prosthetic or osteotomy knees, or other diseases that influence the CBT, such as osteometabolic diseases, except for primary osteoporosis.

**Fig. 1:**
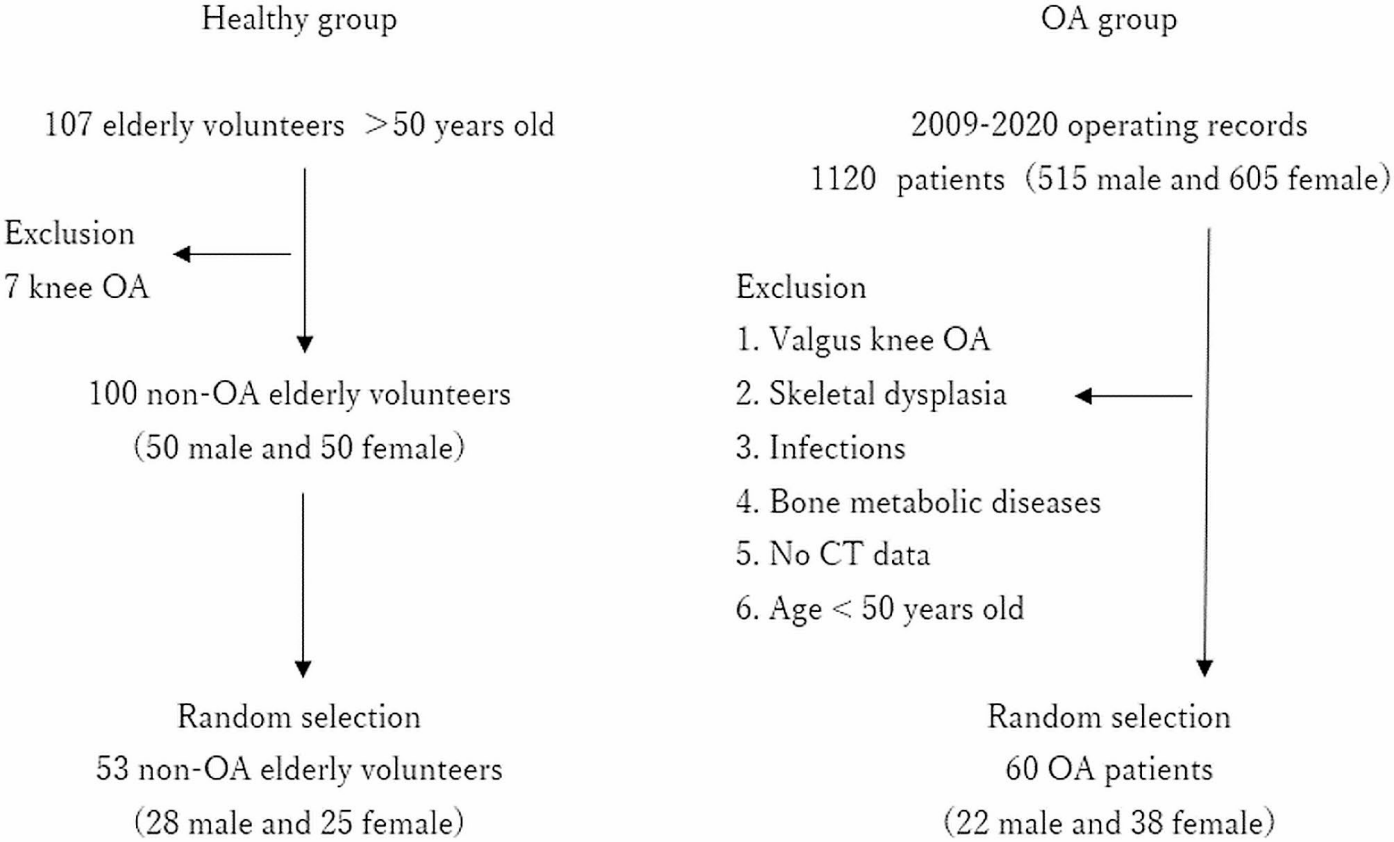
Inclusion and exclusion criteria of the subjects OA: knee osteoarthritis, BMI: body mass index

For the healthy subjects, a total of 107 elderly Japanese volunteers who had no knee complaints or histories of joint disease or major injury in the lower extremity were publicly recruited. The volunteers did not have any competing interests and were not paid any fees. Physicians assessed their general and lower extremity conditions using physical tests and radiographs and excluded seven subjects with radiographic evidence of knee OA. Out of 100 healthy elderly patients (50 males and 50 females) with grades 0–1 according to the Kellgren-Lawrence (K-L) classification [14] and the absence of radiographic knee OA, 53 elderly (aged > 50 years) Japanese volunteers (28 males and 25 females) were randomly selected for this study [Fig. 1]. Two orthopedic surgeons (graders), who were not provided with any clinical information on the patients, performed the K-L classification. When the same subject was assigned different grades, the graders discussed and determined a common K-L grade.

Patients with varus knee OA (age > 50 years) were initially selected from medical operation records from 2009 to 2020, with 1120 surgical knee OA patients using CT data. Finally, using the above exclusion criteria, the lower extremities of 60 patients with varus knee OA (22 males and 38 females), randomly selected from 1,120 knees aged 50 years or older, were included.

The average age ± standard deviation (SD) (range) of the healthy males, healthy females, OA males and OA females was 71 ± 6 years (61 to 83 years), 68 ± 6 years (60 to 83 years), 74 ± 8 years (54 to 86 years), and 74 ± 7 years (61 to 87 years), respectively. The average BMI ± SD (range) of the healthy males, healthy females, OA males and OA females was 23.0 ± 2.1 kg/m2 (17.6 to 27.0 kg/m2), 20.0 ± 1.6 kg/m2 (17.1 to 23.0 kg/m2), 24.9 ± 2.9 kg/m2 (32.4 to 18.5 kg/m2), and 26.6 ± 3.7 kg/m2 (34.7 to 20.7 kg/m2), respectively (Table 1).

**Table 1:** Demographic data

	OA group		Healthy group		Male vs. Female		OA vs. Healthy group	
	Male (*n* = 22)	Female (*n* = 38)	Male (*n* = 28)	Female (*n* = 25)	OA group	Healthy group	Male group	Female group
	Mean ± SD	Mean ± SD	Mean ± SD	Mean ± SD	*p* value	*p* value	*p* value	*p* value
Age (yr)	73.5 ± 7.6	73.6 ± 6.5	71.3 ± 6.1	67.7 ± 5.5	n.s.	n.s.	n.s.	0.003*
Height (cm)	162.8 ± 6.4	148.9 ± 5.5	166.2 ± 4.6	155.4 ± 5.3	< 0.001*	< 0.001*	n.s.	< 0.001*
Weight (kg)	66.0 ± 8.6	59.0 ± 8.6	63.6 ± 7.3	48.3 ± 5.0	0.005*	< 0.001*	n.s.	< 0.001*
BMI (kg/m^2^)	24.9 ± 2.9	26.6 ± 3.7	23.0 ± 2.1	20.0 ± 1.6	n.s.	0.001*	n.s.	< 0.001*
FTA(°)	186.8 ± 2.7	189.3 ± 4.5	178.5 ± 2.5	175.4 ± 2.1	0.024*	0.006*	< 0.001*	< 0.001*
MCT(°)	12.2 ± 4.1	12.3 ± 4.5	9.3 ± 2.7	6.9 ± 2.3	n.s.	n.s.	0.035*	< 0.001*

OA = knee osteoarthritis; SD = standard deviation; BMI = body mass index; FTA = femorotibial angle; MCT = medial compartment of the proximal tibia; *= < 0.05; n.s. = > 0.05

### CT scanning condition

CT scans with a 1-mm interval in the lower extremities from the femoral head to the ankle joint were performed at two institutions using the Somatom Sensation 16 (Siemens Inc., Munich, Germany) and Canon Aquilion 64 CT scanners (Canon Medical Systems, Tochigi, Japan). The scans were obtained at a tube voltage of 120 kVp and a current of 50–400 mA. The field-of-view, matrix, and pixel parameters were 350–400 mm, 512 × 512, and 0.68–0.78 mm/pixel, respectively. The voxel size ranged from 0.68 × 0.68 × 1.00 mm to 0.78 × 0.78 × 1.00 mm. For CT radiation, the mean dose length product was 896.7 ± 129.9 mGy × cm.

### Calculation of cortical thickness

**Fig. 2:**
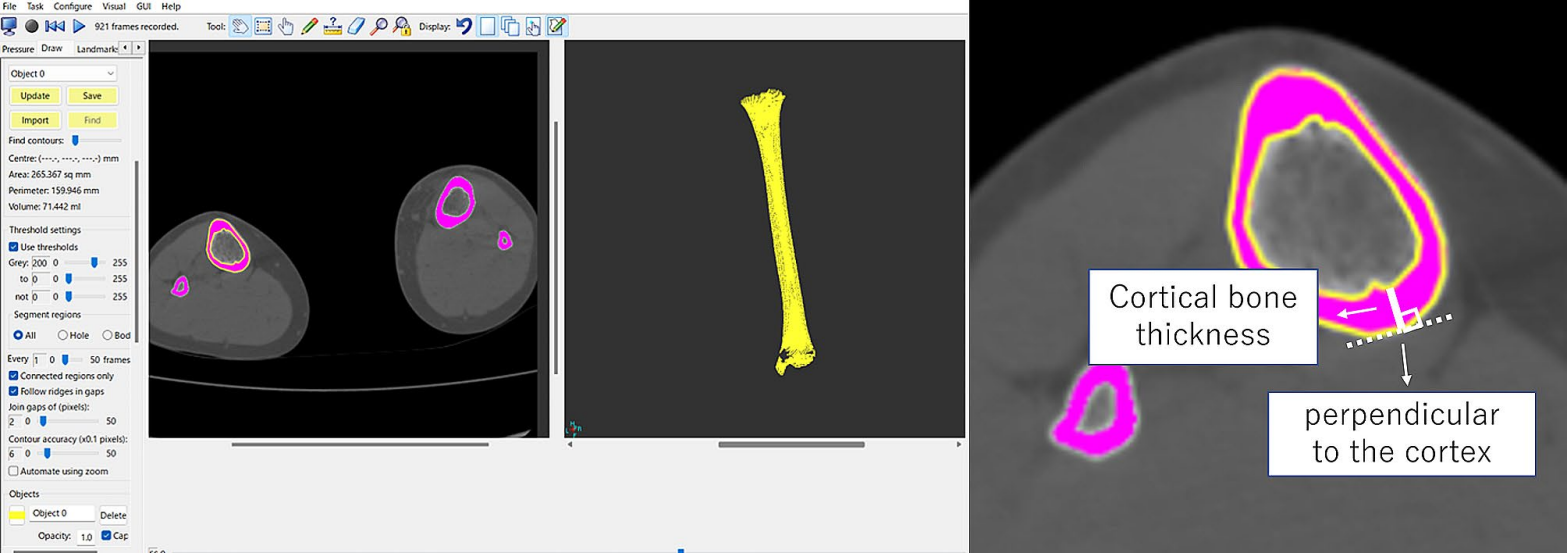
Automatic calculation in a 3D space using the high-resolution cortical thickness measurement from clinical CT data This technique relies on a mathematical model of the anatomy and imaging system that is fitted to data at a large number of sites around the tibia. Given the prior segmentation of the tibial diaphysis, CT values were examined along short lines that straddled and were perpendicular to the cortex

**Fig. 3:**
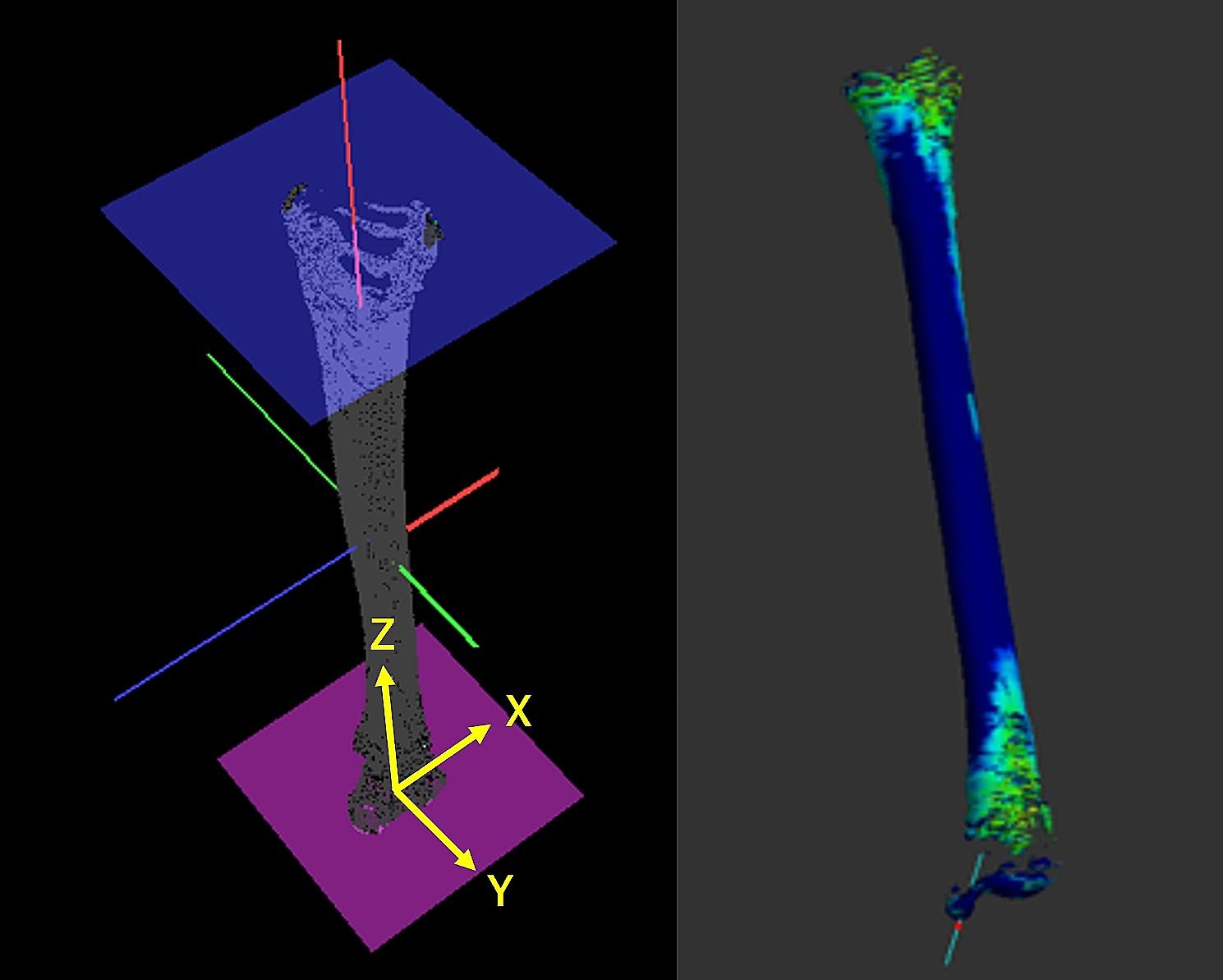
An anatomical coordinate system for the tibia and cortical thickness of the tibia (CBT) mapped using a cortical mapping technique

The CBT of the tibial diaphysis was automatically calculated in 3D space using the high-resolution measurements reported by Treece et al. [2, 3] [Fig. 2], which allowed for accurate estimates of the CBT based on an estimate of cortical density. The technique was implemented using Stradwin software (version 5.3; Medical Imaging Group, Machine Intelligence Laboratory, Cambridge Engineering Department, Cambridge, UK), which is available for free download and is a new tool with demonstrated sub-voxel accuracy in assessing cortical bone properties using routine low-resolution CT. The method uses a complex model-based fit approach with a mathematical model of the anatomy and imaging system, calculates from thousands of data points across the bone surface, and performs assessments using semi-automatic segmentation [2]. The creation of the surface and use of the surface normal to guide the thickness estimation were performed as follows [Fig. 2]: A surface was generated by thresholding the entire dataset and extracting the contours in each plane to subpixel resolution. The contours were then edited locally to correct erroneously excluded regions and remove adjoining structures. A surface was interpolated through these contours, and the surface vertices and normal were used to guide the in-plane thickness estimates using a mathematical equation. The number of measurement points per subject was 2,752–11,296, depending on the tibial length and bone mineral density. The CBT was calculated for each point. Given the prior segmentation of the tibia, the CT values (Hounsfield units) were examined along the short lines that straddled and were perpendicular to the cortex [Fig. 2]. Once the CBT was estimated at each vertex, it was mapped back onto the surface as a color, using a cortical bone-mapping technique [Fig. 3]. A high-resolution thickness map is filtered over the surface.

Regarding accuracy, Treece et al. [2] tested the validity of the constant-density assumption by measuring the true density of cadaveric femurs on high-resolution CT, which had approximately seven times the resolution of low-resolution scans. They reported that the CBT estimates were accurate by up to 0.3 mm. The technique was validated in vivo in the context of osteoporosis and hip fractures within the laminar structures [2, 15].

### Anatomical tibial coordinate system

An anatomical coordinate system for the tibia was constructed using original software [4] [Fig. 3]. First, a three-dimensional CT tibial model was downloaded, and the temporal *z*-axis was defined as the axis connecting the two centers of the approximated circles in the tibial diaphysis in the two transverse planes. Then, the line connecting the attachment of the posterior cruciate ligament to the medial edge of the tibial tuberosity was defined as the *y*-axis (positive anteriorly). The cross product of the temporal *z*-axis and *y*-axis was defined as the tibial *x*-axis (positive right). Finally, the cross-product of the *y*-axis and *x*-axis was the true tibial *z*-axis (positively superior). The origin of the tibial coordinate system was defined as the cross-point between the distal tibial articular surface and the true *z*-axis.

### Evaluation parameters

Twenty-four regions were created by combining six heights (most proximal, 63–70%; proximal, 57–63%; central proximal, 50–57%; central distal, 43–50%; distal, 37–43%; and most distal, 30–37%) and four areas of the axial plane (xy-plane) at 90° (medial, anterior, lateral, and posterior). Height was defined as follows: The tibial length was defined from the midpoint of the tibial eminences to the midpoint of the medial and lateral points on top of the talar dome, representing 100% of the tibial length. The tibial diaphysis was defined as 30–70%, divided into six heights, and categorized as 6–7% (most proximal, 63–70%; proximal, 57–63%; central proximal, 50–57%; central distal, 43–50%; distal, 37–43%; and most distal, 30–37%). Each of the 24 regions (six heights × four areas in the xy-plane) comprised cortical thickness data from 20 to 948 points. The assessment parameter was the average cortical thickness from 20 to 948 points in each region of the tibial diaphysis, divided by the height and area. The cortical thickness in each of the 24 regions was compared among the four groups categorized by sex and OA (OA males, OA females, healthy males, and healthy females). When the data were compared, standardized values rather than actual values were applied. Standardized values divided by tibial length (CBT/tibial length) were applied because the CBT is influenced by body constitution (body weight and height). To standardize the values, because the units of the values must be identical, the tibial length proportional to body height, not body weight, was selected.

### Precision and reproducibility through all processes

To ensure the precision and test–retest reliability of the measurement of CBT in 3D space through all processes, two researchers performed two measurements as one set on 10 subjects randomly selected from each group. CT was performed once for each subject, so that the precision in this study excluded the CT scanning conditions. The precision and reproducibility of the CBT of the total diaphysis were calculated [4]. The mean differences and 95% confidence intervals (Cis) of the differences in the standardized CBT of the total diaphysis were calculated. The mean and maximum differences were 0.1 × 10^− 3^ and 0.3 × 10^− 3^ for researcher #1 and 0.01 × 10^− 3^ and 0.03 × 10^− 3^ for researcher #2, respectively. The 95% CI of the differences was 0.0 × 10^− 3^–0.1 × 10^− 3^ for researcher #1 and 0.0–0.02 × 10^− 3^ for researcher #2, respectively. In the test–retest reliability (SPSS version 21, SPSS Inc., Chicago, IL, USA), intraobserver reproducibility via the intraclass correlation coefficient of the two measurements was 0.925 (*p* < 0.001) for researcher #1 and 0.998 (*p* < 0.001) for researcher #2. Inter-observer reproducibility via the interclass correlation coefficient was 0.989 (*p* = 0.001).

### Three-dimentional lower extremity alignment assessment system

**Fig. 4:**
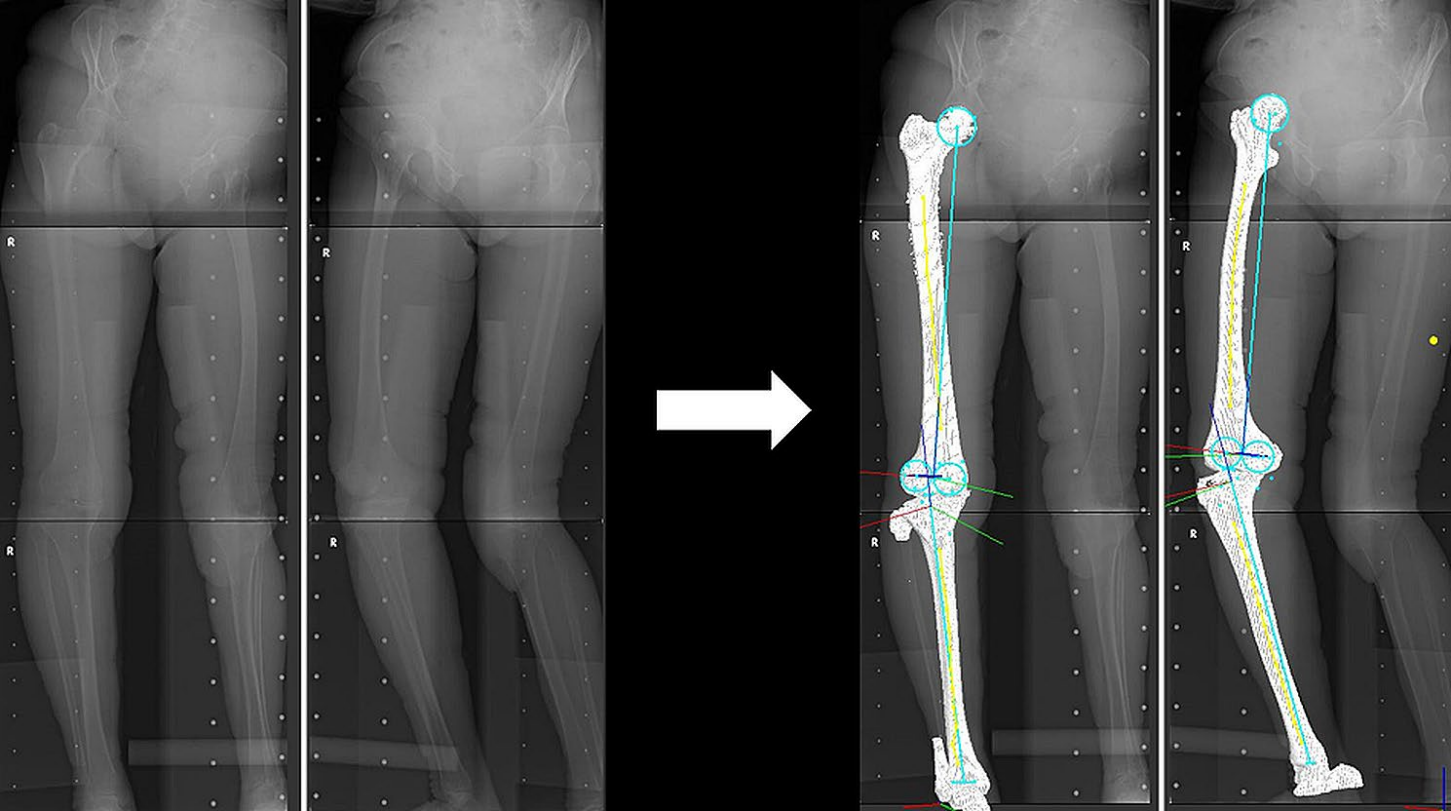
Three-dimensional lower extremity alignment assessment system Thirty-four skin markers were attached to the subjects. For the ten shank markers and 12 thigh markers, the original marker included a steel ball to detect its 2D position on X-ray images. The 3D position of the femorotibial bones can be estimated by superimposing 3D skeletal models onto the bony outline of the lower extremity under weight-bearing conditions using a 3D-to-2D image registration technique

A 3D lower-extremity alignment assessment system (Knee CAS, LEXI Inc., Tokyo, Japan) based on biplanar long-leg X-rays was developed to assess lower-extremity alignment and bone morphology. This system uses a 3D to 2D image registration technique [6, 7] and enables automatic, strict measurement of all parameters under weight-bearing conditions with high accuracy in 3D space [6] [Fig. 4]. A stereophotogrammetric X-ray apparatus consisting of a 0–60° turn stage was used. The 3D position of the femorotibial bones can be estimated by superimposing 3D skeletal models onto the bony outline of the lower extremities under weight-bearing conditions [6] [Fig. 4]. The femoral and tibial coordinate systems constructed in the 3D skeletal model were determined as previously described [4, 7] [Fig. 4]. The overlapping procedure used the 3D to 2D image registration technique, with a matching error within a range of 0.68 mm in rotation and 0.5 mm in translation [6].

**Fig. 5:**
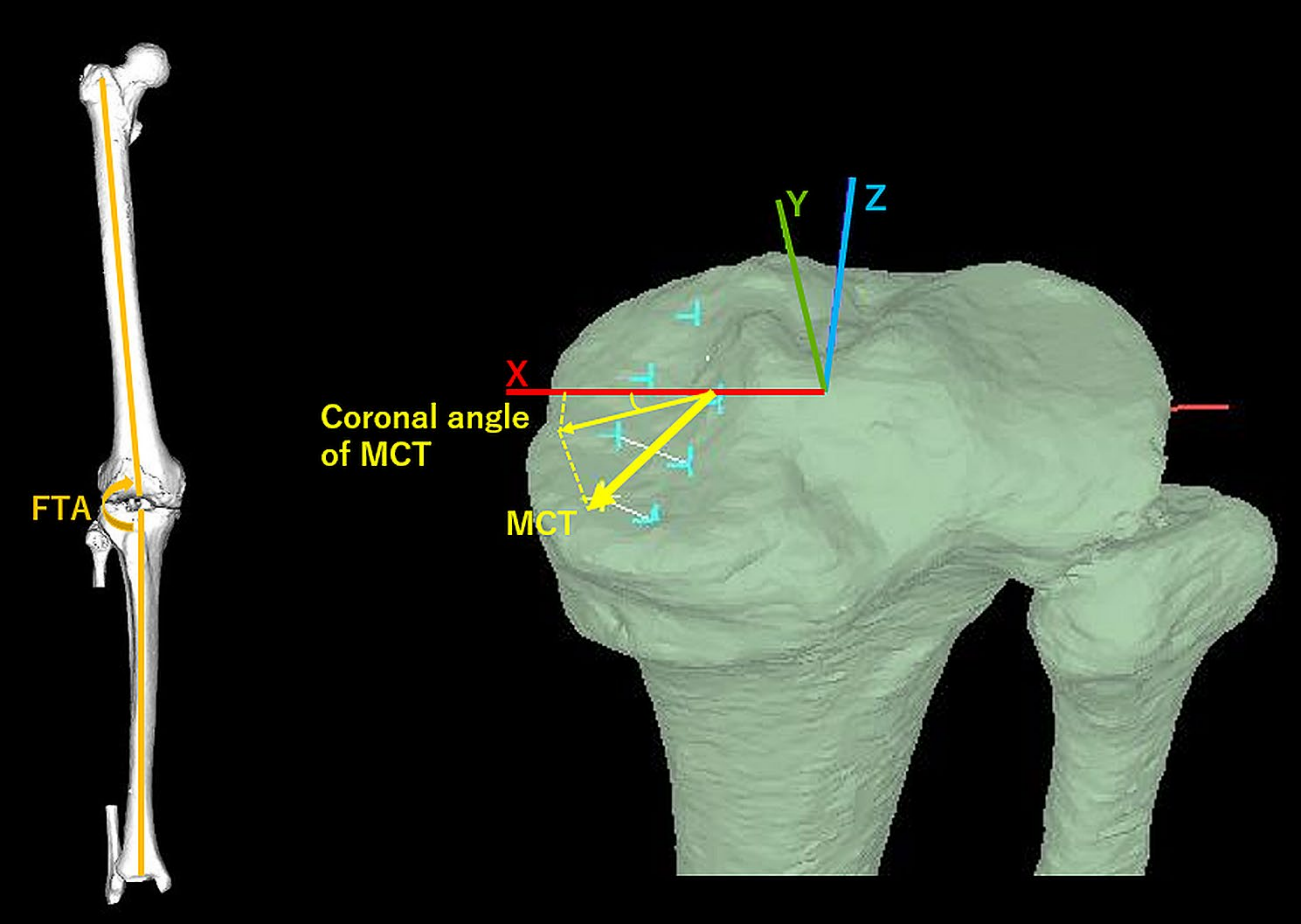
Femorotibial angle (FTA) and best-fitting “approximation plane” in the medial compartment of the proximal tibia (MCT) definition The FTA was the angle between the femoral and tibial anatomical axes projected onto the coronal plane in the femoral coordinate system. Schematic diagram shows that the MCT, and coronal angle of the MCT. The minimum angle in 3D space between the *x*-axis of the tibial coordinate system and the crossing line consisted of the xz-plane of the tibial coordinate system and the approximation plane of the MCT, which is defined as the coronal angle of the MCT.

In terms of the femorotibial angle (FTA), anatomical reference axes were determined in 3D space to evaluate the true 3D lower extremity alignment. A point-group centroid was automatically calculated for the ten respective cross-sectional planes, which divided the femoral diaphysis into 11 equal sections using the femoral coordinate system. The same calculation was performed for 12 cross-sectional planes that divided the tibial diaphysis into 13 equal sections in the tibial coordinate system. The anatomical axes were defined as regression lines obtained by approximating the distances from these ten centroids in the femur and 12 centroids in the tibia using the least-squares method. The FTA is defined as the angle between the femoral and tibial anatomical axes projected onto the coronal plane in the femoral coordinate system [Fig. 5]. A larger FTA indicated a larger varus alignment.

### Approximation plane of the MCT

The best-fitting “approximation plane” in the MCT was determined by the least-squares method, using eight points digitized on the MCT [8] [Fig. 5]. The least-squares method is an established approach in regression analysis used to approximate solutions for overestimated systems. The digitization points did not include osteophytes or large deformities such as excessive concavity to obtain high precision and reproducibility. The approximation plane of the MCT and the normal vector were mathematically calculated. The angle between the normal vector and each axis was the minimum angle in 3D space between the *x*-axis of the tibial coordinate system and the crossing line consisting of the approximation plane of the MCT and the xz-plane of the tibial coordinate system, defined as the coronal angle of the MCT [Fig. 5]. These MCT angles were not projected on each plane in 2D space but were defined in 3D space. The MCT morphology is a clinical interpretation. Smaller angles for each parameter indicated a greater inclination toward each axis of the tibial coordinate system. The coronal angles of the MCT were used as assessment parameters. The mean and maimum differences were set as 1.1° for precision. The intra- and interobserver reproducibilities, expressed as intra- and interclass correlation coefficients, were 0.958 and 0.893, respectively [8].

### Statistical analyses

The actual and standardized values are listed in Tables 2 and 3, respectively. In Table 4, the standardized cortical thicknesses at each height (total, most proximal, proximal, central proximal, central distal, distal, and most distal diaphysis) of the four groups (OA male, OA female, healthy male, and healthy female) were compared among the four areas of the transverse plane (medial, anterior, lateral, and posterior areas) using repeated measures ANOVA with Tukey’s test or Friedman’s test as the counterpart of repeated measures ANOVA. In Table 5, the standardized cortical thickness in each of the 24 regions is compared among the four groups categorized by sex and age (OA male, OA female, healthy male, and healthy female) using one-way ANOVA with post-hoc or Kruskal-Wallis tests as the nonparametric equivalent of ANOVA. In Table 6, the medial/lateral (M/L) and anterior/posterior (A/P) ratios at each height (total, most proximal, proximal, central proximal, central distal, distal, and most distal diaphysis) of the four groups (OA male, OA female, healthy male, and healthy female) were compared using one-way ANOVA with a post-hoc test or Kruskal-Wallis test as the nonparametric equivalent of ANOVA. Pearson’s product moment correlation coefficient or Spearman’s rank correlation coefficient was applied, depending on the Shapiro–Wilk test (Tables 7 and 8). Correlations between FTA (Table 7), MCT (Table 8), and standardized thickness at each height (total, most proximal, proximal, central proximal, central distal, distal, and most distal diaphysis) in the four groups (OA male, OA female, healthy male, and healthy female) were compared.

**Table 2:** Cortical thickness in tibia diaphysis (actual values)

Actual values (mm)	OA group (*n* = 60)				Healthy group (*n* = 53)			
	Male (*n* = 22)		Female (*n* = 38)		Male (*n* = 28)		Female (*n* = 25)	
	mean	95%CI	mean	95%CI	mean	95%CI	mean	95%CI
Total diaphysis								
Medial	5.9	5.5–6.3	5.3	5.1–5.5	5.6	5.4–5.9	5.4	5.2–5.6
Lateral	5.5	5.3–5.7	5.2	5.0-5.3	5.7	5.5–5.9	5.6	5.5–5.8
Anterior	7.8	7.2–8.3	6.6	6.2-7.0	7.9	7.5–8.3	7.3	7.0-7.6
Posterior	5.2	4.9–5.4	4.6	4.4—4.7	5.3	5.1–5.5	5.0	4.8–5.1
Most proximal diaphysis								
Medial	5.7	5.4-6.0	5.4	5.1–5.6	5.5	5.2–5.8	5.3	5.1–5.6
Lateral	4.9	4.7–5.1	4.8	4.7-5.0	5.5	5.3–5.7	5.5	5.3–5.7
Anterior	6.8	6.3–7.3	6.3	5.8–6.7	7.5	7.0–8.0	7.3	7.0-7.7
Posterior	4.8	4.7-5.0	4.7	4.5–4.9	5.1	5.0-5.3	5.2	4.9–5.4
Proximal diaphysis								
Medial	5.6	5.2-6.0	5.4	5.2–5.7	5.6	5.4–5.9	5.2	4.9–5.5
Lateral	5.3	5.0-5.6	5.0	4.8–5.2	5.7	5.4–5.9	5.6	5.4–5.9
Anterior	7.1	6.5–7.7	6.1	5.7–6.4	7.2	6.9–7.5	6.9	6.5–7.2
Posterior	5.1	4.9–5.3	4.7	4.5–4.9	5.4	5.2–5.6	5.0	4.8–5.2
Central proximal diaphysis								
Medial	5.6	5.1-6.0	5.2	5.0-5.5	5.4	5.0-5.8	5.5	5.2–5.8
Lateral	5.3	5.1–5.6	5.2	5.0-5.4	5.7	5.3–6.1	5.6	5.4–5.9
Anterior	8.0	7.3–8.7	6.5	6.0–7.0	7.9	7.5–8.4	7.5	7.1–7.9
Posterior	5.2	4.9–5.5	4.6	4.4–4.8	5.2	5.0-5.4	4.8	4.7-5.0
Central distal diaphysis								
Medial	6.3	5.8–6.8	5.3	5.0-5.6	5.8	5.4–6.2	5.4	4.9–5.8
Lateral	5.7	5.4–5.9	5.4	5.2–5.6	5.8	5.5-6.0	5.7	5.4-6.0
Anterior	8.8	8.0-9.5	7.1	6.6–7.6	8.9	8.3–9.5	7.6	7.2-8.0
Posterior	5.3	5.0-5.6	4.4	4.3–4.6	5.4	5.1–5.6	4.8	4.6-5.0
Distal diaphysis								
Medial	6.6	6.0-7.3	5.4	5.1–5.7	6.0	5.6–6.4	5.3	4.9–5.7
Lateral	6.1	5.7–6.4	5.3	5.0-5.6	5.9	5.7–6.1	5.8	5.6-6.0
Anterior	8.8	8.1–9.5	7.3	6.8–7.8	9.1	8.4–9.7	7.7	7.2–8.2
Posterior	5.5	5.2–5.8	4.4	4.3–4.6	5.5	5.2–5.8	4.8	4.7–4.9
Most distal diaphysis								
Medial	6.4	5.6–7.2	5.2	4.9–5.5	5.8	5.4–6.2	5.8	5.4–6.3
Lateral	6.4	5.9–6.9	5.3	5.0-5.6	6.2	5.8–6.7	5.9	5.6–6.1
Anterior	8.3	7.6-9.0	6.9	6.5–7.2	8.7	8.0-9.3	7.6	7.1–8.1
Posterior	5.3	5.0-5.6	4.4	4.2–4.6	5.6	5.3–5.9	4.8	4.6-5.0

OA = knee osteoarthritis; 95%CI = 95% confidence interval

**Table 3:** Cortical thickness in tibia diaphysis (standardized values)

Standardized values (×10^− 3^)	OA group (*n* = 60)				Healthy group (*n* = 53)			
	Male (*n* = 22)		Female (*n* = 38)		Male (*n* = 28)		Female (*n* = 25)	
	mean	95%CI	mean	95%CI	mean	95%CI	mean	95%CI
Total diaphysis								
Medial	17.9	16.7–19.1	17.5	16.8–18.1	17.1	16.4–17.7	17.4	16.6–18.2
Lateral	16.6	15.8–17.4	16.9	16.3–17.6	17.3	16.6–18.0	18.1	17.5–18.7
Anterior	23.5	21.8–25.1	21.6	20.3–22.8	23.9	22.7–25.1	23.5	22.5–24.5
Posterior	15.6	14.9–16.2	15.0	14.5–15.5	16.1	15.6–16.5	16.0	15.5–16.5
Most proximal diaphysis								
Medial	17.3	16.3–18.2	17.6	16.9–18.3	16.8	15.8–17.7	17.1	16.3–17.9
Lateral	14.9	14.2–15.6	15.9	15.3–16.6	16.8	16.1–17.5	17.6	16.9–18.4
Anterior	20.4	18.9–21.9	20.6	19.1–22.0	22.7	21.2–24.3	23.5	22.4–24.6
Posterior	14.6	14.1–15.1	15.5	14.8–16.2	15.6	15.1–16.1	16.7	15.7–17.6
Proximal diaphysis								
Medial	16.8	15.6–18.0	17.8	16.9–18.6	17.1	16.3–18.0	16.8	15.6–17.9
Lateral	16.0	15.0-16.9	16.4	15.7–17.0	17.1	16.3–18.0	18.2	17.3–19.1
Anterior	21.5	19.7–23.2	19.9	18.8–21.1	21.9	20.9–22.9	22.2	21.0-23.4
Posterior	15.3	14.7–16.0	15.4	14.7–16.0	16.3	15.6–16.9	16.1	15.4–16.9
Central proximal diaphysis								
Medial	16.8	15.5–18.1	17.2	16.4–18.0	16.4	15.3–17.5	17.6	16.6–18.6
Lateral	16.1	15.2–16.9	17.0	16.3–17.8	17.2	15.9–18.5	18.2	17.2–19.2
Anterior	24.2	22.2–26.2	21.4	19.9–22.8	24.0	22.7–25.3	24.1	22.9–25.3
Posterior	15.7	14.8–16.6	15.2	14.6–15.9	15.8	15.2–16.4	15.5	15.0–16.0
Central distal diaphysis								
Medial	19.1	17.4–20.7	17.5	16.5–18.4	17.4	16.2–18.6	17.4	15.8–18.9
Lateral	17.1	16.1–18.0	17.7	16.9–18.6	17.5	16.6–18.4	18.4	17.3–19.5
Anterior	26.4	24.3–28.5	23.2	21.5–25.0	27.0	25.2–28.9	24.4	23.2–25.7
Posterior	15.9	15.0-16.8	14.5	14.0–15.0	16.2	15.5–16.9	15.4	14.8–16.1
Distal diaphysis								
Medial	20.1	18.1–22.1	17.7	16.7–18.6	18.1	16.9–19.3	17.2	15.8–18.7
Lateral	18.3	17.1–19.5	17.6	16.5–18.6	18.0	17.2–18.7	18.7	18.0-19.4
Anterior	26.6	24.5–28.8	23.9	22.3–25.5	27.5	25.6–29.4	24.6	23.1–26.1
Posterior	16.7	15.8–17.5	14.5	14.0-15.1	16.6	15.9–17.3	15.5	15.0–16.0
Most Distal diaphysis								
Medial	19.3	16.7–21.8	16.9	16.0-17.8	17.5	16.3–18.8	18.9	17.3–20.4
Lateral	19.4	17.8–20.9	17.4	16.5–18.3	18.9	17.7–20.0	19.0	18.1–19.9
Anterior	25.1	23.0-27.3	22.6	21.4–23.7	26.2	24.3–28.1	24.5	22.9–26.1
Posterior	16.1	15.2–17.0	14.4	13.8–15.0	16.9	16.2–17.5	15.5	14.9–16.1

OA = knee osteoarthritis; 95%CI = 95% confidence interval; standardized values mean the actual values divided by the tibia length

**Table 4:** Comparison between medial and lateral or between anterior and posterior standardized thickness

		OA male		OA female		Healthy male		Healthy female	
		*p* value	Summary	*p* value	Summary	*p* value	Summary	*p* value	Summary
Total diaphysis	M–L	0.001*	M > L	n.s.	—	n.s.	—	n.s.	—
	A–P	< 0.001*	A > P	< 0.001*	A > P	< 0.001*	A > P	< 0.001*	A > P
Most proximal diaphysis	M–L	< 0.001*	M > L	< 0.001*	M > L	n.s.	–	n.s.	—
	A–P	< 0.001*	A > P	< 0.001*	A > P	< 0.001*	A > P	< 0.001*	A > P
Proximal diaphysis	M–L	n.s.	–	0.001*	M > L	n.s.	–	0.008*	M < L
	A–P	< 0.001*	A > P	< 0.001*	A > P	< 0.001*	A > P	< 0.001*	A > P
Central proximal diaphysis	M–L	n.s.	–	n.s.	–	n.s.	–	n.s.	–
	A–P	< 0.001*	A > P	< 0.001*	A > P	< 0.001*	A > P	< 0.001*	A > P
Central distal diaphysis	M–L	0.008*	M > L	n.s.	–	n.s.	–	n.s.	–
	A–P	< 0.001*	A > P	< 0.001*	A > P	< 0.001*	A > P	< 0.001*	A > P
Distal diaphysis	M–L	0.023*	M > L	n.s.	–	n.s.	–	0.049*	M < L
	A–P	< 0.001*	A > P	< 0.001*	A > P	< 0.001*	A > P	< 0.001*	A > P
Most distal diaphysis	M–L	n.s.	–	n.s.	–	0.026*	M < L	n.s.	–
	A–P	< 0.001*	A > P	< 0.001*	A > P	< 0.001*	A > P	< 0.001*	A > P

The standardized cortical thickness in each height (most proximal, proximal, central proximal, central distal, distal and most distal diaphysis) of the four groups (OA male, OA female, healthy male, and healthy female) was compared among the four areas of the axial plane (medial, anterior, lateral, and posterior areas), using repeated measures ANOVA with Tukey test or the Friedman’s test as the counterpart of repeated measures ANOVA. OA = knee osteoarthritis; M–L = comparison between medial and lateral standardized thickness; A–*P* = comparison between anterior and posterior standardized thickness; M = medial standardized thickness; L = lateral standardized thickness; A = anterior standardized thickness; *P* = posterior standardized thickness; *= < 0.05; n.s. = > 0.05

**Table 5:** Sex– and OA– related differences

		Male (M) vs. Female (F)				OA (O) vs. Healthy group (H)			
		OA group		Healthy group		Male group		Female group	
		*p* value	Summary	*p* value	Summary	*p* value	Summary	*p* value	Summary
Total diaphysis	M	n.s.	—	n.s.	–	n.s.	—	n.s.	—
	L	n.s.	—	n.s.	–	n.s.	—	0.043*	OA < H
	A	n.s.	—	n.s.	–	n.s.	—	n.s.	–
	*P*	n.s.	—	n.s.	–	n.s.	—	0.031*	OA < H
Most proximal diaphysis	M	n.s.	-	n.s.	–	n.s.	—	n.s.	—
	L	n.s.	–	n.s.	—	0.003*	OA < H	0.008*	OA < H
	A	n.s.	—	n.s.	–	n.s.	—	0.018*	OA < H
	*P*	n.s.	–	n.s.	—	0.021*	OA < H	n.s.	-
Proximal diaphysis	M	n.s.	-	n.s.	–	n.s.	—	n.s.	—
	L	n.s.	–	n.s.	—	n.s.	-	0.006*	OA < H
	A	n.s.	—	n.s.	–	n.s.	—	0.037*	OA < H
	*P*	n.s.	–	n.s.	—	n.s.	–	n.s.	-
Central proximal diaphysis	M	n.s.	–	n.s.	–	n.s.	–	n.s.	–
	L	n.s.	–	n.s.	–	n.s.	–	n.s.	–
	A	n.s.	–	n.s.	—	n.s.	—	0.037*	OA < H
	*P*	n.s.	–	n.s.	—	n.s.	–	n.s.	—
Central distal diaphysis	M	n.s.	–	n.s.	–	n.s.	–	n.s.	–
	L	n.s.	–	n.s.	–	n.s.	–	n.s.	–
	A	n.s.	-	n.s.	—	n.s.	—	n.s.	—
	*P*	0.013*	M > F	n.s.	—	n.s.	–	n.s.	—
Distal diaphysis	M	0.047*	M > F	n.s.	–	n.s.	—	n.s.	–
	L	n.s.	—	n.s.	–	n.s.	–	n.s.	–
	A	n.s.	-	n.s.	–	n.s.	—	n.s.	–
	*P*	< 0.001*	M > F	n.s.	-	n.s.	–	n.s.	–
Most distal diaphysis	M	n.s.	—	n.s.	–	n.s.	—	n.s.	–
	L	n.s.	—	n.s.	–	n.s.	–	n.s.	–
	A	n.s.	–	n.s.	–	n.s.	—	n.s.	–
	*P*	0.005*	M > F	0.049*	M > F	n.s.	–	n.s.	–

The standardized cortical thickness in each of the 24 regions was compared among the four groups categorized by sex and age (OA male, OA female, healthy male, and healthy female), using one-way ANOVA with post hoc or Kruskal-Wallis test as the nonparametric equivalent of ANOVA. OA = knee osteoarthritis; M = medial thickness; L = lateral thickness; A = anterior thickness; *P* = posterior thickness; *= < 0.05; n.s. = > 0.05

**Table 6:** Comparison of M/L ratio and A/P ratio between each group

		Male (M) vs. Female (F)				OA (O) vs. Healthy group (H)			
		OA group		Healthy group		Male group		Female group	
		*p* value	Summary	*p* value	Summary	*p* value	Summary	*p* value	Summary
Total diaphysis	M/L	n.s.	—	n.s.	–	0.015*	OA > H	0.046*	OA > H
	A/P	n.s.	—	n.s.	–	n.s.	—	n.s.	–
Most proximal diaphysis	M/L	n.s.	-	n.s.	–	0.007*	OA > H	0.002*	OA > H
	A/P	n.s.	—	n.s.	–	n.s.	—	n.s.	—
Proximal diaphysis	M/L	n.s.	-	n.s.	–	n.s.	—	0.001*	OA > H
	A/P	n.s.	—	n.s.	–	n.s.	—	n.s.	—
Central proximal diaphysis	M/L	n.s.	–	n.s.	–	n.s.	–	n.s.	–
	A/P	n.s.	–	n.s.	—	n.s.	—	0.018*	OA < H
Central distal diaphysis	M/L	n.s.	–	n.s.	–	n.s.	–	n.s.	–
	A/P	n.s.	-	n.s.	—	n.s.	—	n.s.	—
Distal diaphysis	M/L	n.s.	-	n.s.	–	n.s.	—	n.s.	–
	A/P	n.s.	-	n.s.	–	n.s.	—	n.s.	–
Most distal diaphysis	M/L	n.s.	—	n.s.	–	n.s.	—	n.s.	–
	A/P	n.s.	-	n.s.	–	n.s.	—	n.s.	–

M/L = medial/lateral ratio; A/*P* = anterior/posterior ratio; OA = knee osteoarthritis; *= < 0.05; n.s. = > 0.05

**Table 7:** Correlations between FTA and standardized thickness

		OA male		OA female		Healthy male		Healthy female	
		CC	*p* value	CC	*p* value	CC	*p* value	CC	*p* value
Total diaphysis	M	0.278	—	0.264	–	0.11	—	0.107	—
	L	0.134	—	0.235	–	-0.075	—	-0.02	–
	A	0.068	—	-0.004	–	-0.185	—	-0.037	–
	*P*	0.097	—	0.191	–	-0.054	—	-0.139	–
Most proximal diaphysis	M	0.213	—	0.365	0.026*	-0.112	—	-0.131	—
	L	0.261	–	0.126	—	0.324	–	-0.079	–
	A	0.17	—	0.124	–	-0.244	—	-0.026	–
	*P*	0.188	–	0.162	—	-0.144	–	-0.178	–
Proximal diaphysis	M	0.294	–	0.369	0.023*	0.371	—	0.015	—
	L	0.23	–	0.188	—	0.031	–	-0.023	–
	A	-0.034	—	0.111	–	-0.191	—	-0.06	–
	*P*	0.212	–	0.243	—	-0.041	–	-0.25	–
Central proximal diaphysis	M	0.087	–	0.257	–	0.463	0.013*	0.289	–
	L	0.132	–	0.052	–	0.103	–	0.051	–
	A	-0.069	–	-0.002	—	-0.028	—	-0.172	–
	*P*	-0.009	–	0.021	—	0.14	–	-0.195	—
Central distal diaphysis	M	0.147	–	0.143	-	-0.002	–	0.341	–
	L	0.042	–	0.337	0.038*	0.198	–	-0.089	–
	A	0.013	–	-0.103	—	-0.041	—	0.05	—
	*P*	0.039	–	0.255	—	-0.179	–	-0.051	—
Distal diaphysis	M	0.273	–	-0.084	–	0.011	—	0.228	–
	L	-0.034	—	0.354	0.029*	0.022	–	0.13	–
	A	0.142	–	-0.172	–	-0.108	—	-0.09	–
	*P*	-0.055	–	0.295	–	0.005	–	0.144	–
Most distal diaphysis	M	0.283	—	0.185	–	-0.193	—	-0.421	0.036*
	L	-0.112	—	-0.082	–	-0.01	–	0.211	–
	A	0.238	–	-0.146	–	-0.197	—	0.156	–
	*P*	0.155	–	-0.01	–	0.014	–	0.3	–

FTA = femorotibial angle; CC = correlation coefficient; OA = knee osteoarthritis; M = medial thickness; L = lateral thickness; A = anterior thickness; *P* = posterior thickness; *= < 0.05; n.s. = > 0.05

**Table 8:** Correlations between MCT and standardized thickness

		OA male		OA female		Healthy male		Healthy female	
		CC	*p* value	CC	*p* value	CC	*p* value	CC	*p* value
Total diaphysis	M	0.358	—	0.409	0.011*	-0.049	—	0.032	—
	L	0.166	—	0.398	0.013*	-0.078	—	0.007	–
	A	0.125	—	0.13	–	0.063	—	-0.013	–
	*P*	0.263	—	0.273	–	0.04	—	-0.078	–
Most proximal diaphysis	M	0.594	0.004*	0.554	< 0.001*	-0.211	—	0.164	—
	L	0.245	–	0.066	—	-0.154	–	0.048	–
	A	0.503	0.017*	0.271	–	-0.043	—	0.039	–
	*P*	0.227	–	0.124	—	0.189	–	-0.155	–
Proximal diaphysis	M	0.61	0.003*	0.368	0.023*	0.094	—	0.293	—
	L	0.032	–	0.267	—	-0.148	–	0.096	–
	A	0.204	—	0.204	–	0.038	—	0.024	–
	*P*	0.163	–	0.290	—	-0.132	–	0.015	–
Central proximal diaphysis	M	0.416	–	0.375	0.020*	0.188	–	0.006	–
	L	-0.045	–	0.198	–	0.06	–	-0.057	–
	A	0.165	–	0.112	—	0.208	—	-0.349	–
	*P*	0.273	–	0.271	—	0.084	–	-0.121	—
Central distal diaphysis	M	0.151	–	0.380	0.019*	0.012	–	0.044	–
	L	0.03	–	0.408	0.011*	0.182	–	-0.052	–
	A	-0.038	–	0.021	—	0.112	—	-0.004	—
	*P*	0.279	–	0.391	0.015*	-0.01	–	-0.369	—
Distal diaphysis	M	0.052	–	0.175	–	-0.111	—	-0.145	–
	L	0.185	—	0.448	0.005*	-0.227	–	-0.066	–
	A	-0.126	–	-0.078	–	0.034	—	0.102	–
	*P*	0.26	–	0.304	–	0.035	–	0.082	–
Most distal diaphysis	M	0.123	—	0.232	–	-0.051	—	-0.299	–
	L	0.271	—	0.307	–	-0.117	–	0.068	–
	A	0.184	–	0.029	–	0.027	—	0.21	–
	*P*	0.123	–	0.07	–	-0.025	–	0.105	–

MCT = medial compartment of the proximal tibia; CC = correlation coefficient; OA = knee osteoarthritis; M = medial thickness; L = lateral thickness; A = anterior thickness; *P* = posterior thickness; *= < 0.05; n.s. = > 0.05

Statistical significance was set at a *p*-value < 0.05 (SPSS version 21, SPSS Inc., Chicago, IL, USA).

A sample size calculation was performed to determine the main outcome of the correlation between MCT inclination and CBT of the most proximal-medial region in OA males and OA females. The sample size calculation used the following conditions: α error: 0.05; 1-β error: 0.80; correlation coefficient: OA male, 0.594; OA female, 0.554. Nineteen tibias in OA males and 23 tibias in OA females were needed to analyze the main outcomes. This study had a sufficient sample size of OA males and females with significant differences (22 tibias in OA males and 38 tibias in OA females).

## Results

In the comparison of the FTA and MCT between the healthy and OA groups, FTA and MCT were significantly higher in both OA males and OA females (FTA: male, *p* < 0.001; female, *p* < 0.001; MCT: male, *p* = 0.035; female, *p* < 0.001) (Table 1). The FTA was significantly higher in females with OA than in males with OA (*p* = 0.024), whereas it was significantly higher in healthy males than in healthy females (*p* = 0.06).

The healthy group showed a trend of thicker CBT in the lateral areas than in the medial areas in the mediolateral comparison within each group as the structural characteristics [male: most distal (*p* = 0.026); female: proximal (*p* = 0.008), distal (*p* = 0.049)] (Tables 2, 3 and 4). In contrast, the OA group showed a thicker CBT in the medial areas than in the lateral areas from the most proximal to the central proximal height [OA males: total (*p* = 0.001), most proximal (*p* < 0.001), central distal (*p* = 0.008), distal (*p* = 0.023); OA females: most proximal (*p* < 0.001), proximal (*p* = 0.001)].

In terms of sex differences in each region (Table 5), the healthy group demonstrated no sex differences in most regions, whereas the OA groups had sex differences, in that males showed thicker CBT than females, mainly at the distal heights.

In the comparison between the healthy and OA groups, males showed no significant differences in most regions. In females, the lateral CBT from the proximal to the center height was thinner in females with OA than in healthy females. The medial CBT in the proximal tibia was equal to or thicker than that in females with OA. As a basic trend, the CBT in females with OA was thinner than that in healthy females, whereas the medial CBT was equal to or thicker in females with OA.

No sex differences were found in the M/L or A/P ratios (Table 6) in either the healthy or OA groups. In the comparison between the healthy and OA groups in the M/L and A/P ratios, the M/L ratio was significantly higher for both OA males and OA females in the total and most proximal heights.

Regarding the correlation between FTA and CBT, there was no significant difference in OA males (Table 7), but OA females showed a significant weak positive correlation in four regions [most proximal-medial (CC = 0.365, *p* = 0.026): proximal-medial (CC = 0.369, *p* = 0.023), central distal-lateral (CC = 0.337, *p* = 0.038), and distal-lateral (CC = 0.354, *p* = 0.029)]. No significant differences were found in most regions in the healthy group.

Regarding the correlation between MCT inclination and CBT (Table 8), males with OA showed significant positive correlations in the proximal-medial regions [most proximal-medial (CC = 0.594, *p* = 0.004) and proximal-medial (CC = 0.61, *p* = 0.003)]. Females with OA showed a significantly positive correlation mainly in the medial areas [total-medial (CC = 0.409, *p* = 0.011), most proximal-medial (CC = 0.554, *p* < 0.001), proximal-medial (CC = 0.368, *p* = 0.023), central proximal-medial (CC = 0.375, *p* = 0.020), and central distal-medial (CC = 0.380, *p* = 0.019)]. No significant correlation was observed in the healthy group.

## Discussion

The most important findings of this study were as follows: (1) the CBT in osteoarthritic knees showed a different trend from that in healthy knees, especially for medial areas; and (2) the medial CBT of the proximal tibia correlated with the FTA and MCT inclination in the OA group.

In terms of sex differences, males with OA tended to be thicker distally than centrally. The CBT at the distal tibia has been reported to correlate with bone mineral density [16]. The prevalence of osteoporosis is higher in females than in males. Yoshimura et al. [17] noted that the prevalence of osteoporosis in Japanese over 80 years of age was 65% in females and 13% in males. Although the relationship between osteoporosis and knee OA remains controversial, Im et al. [18] reported a positive correlation between hip bone mineral density and alignment. Using multiple linear regression, Zang et al. [19] showed a higher prevalence of osteoporosis in knee OA and a significant association between varus deformity of knee OA and bone mineral density of the spine, femoral neck, and hip. Females with OA had lower bone mineral density due to osteoporosis, which may have resulted in a significantly lower CBT at the distal tibia than males with OA in this study. Similarly, osteoporosis was probably responsible for the thinner total CBT of the tibial diaphysis, except for the medial areas, in the OA group than in the healthy group.

In a comparison between OA patients and healthy females, the lateral CBT in the central to proximal tibia was thinner in knee OA. In addition, the medial CBT in the proximal tibia in knee OA is equal to or thicker. The M/L ratio of the proximal tibia was significantly higher in the patients with knee OA. Taking all the above results into account, it can be said that females with OA have equal or thicker medial CBT and thinner lateral CBT in the proximal tibia than healthy females. Compressive stress on the medial compartment has been reported to be higher in osteoarthritic knees with varus malalignment than in healthy knees [20, 21]. Continuous overloading of the medial compartment probably resulted in an increased medial CBT in the proximal tibia as a result of remodeling. In the lateral CBT, whole lower extremity alignment may be involved. It has been known that the bone morphology is determined based on the balance of remodeling between “bone formation by compression” and “bone resorption by extension [22]. In varus alignment, the medial area corresponds to compression and the lateral area corresponds to extension; thus, it makes sense that the medial area would be thicker and the lateral area thinner. In addition, as noted in the preceding paragraph, one of the causes for the thinner lateral CBT can be explained by osteoporosis in knee OA [17, 19]. This study showed that, as a basic trend, especially for females, most areas of the CBT in the OA group were thinner than those in the healthy group due to possible osteoporosis, whereas the medial CBT in varus knee OA was equal or thicker due to the medial load concentration.

The relationship between the proximal-medial CBT and each of the FTA and MCT inclinations showed correlations in both OA females and males, and the MCT had a stronger correlation than the FTA. While whole lower extremity alignment reflects various factors such as cruciate ligaments, cartilage, and muscle strength, the MCT inclination and medial CBT of the proximal tibia are assumed to directly reflect the accumulation of mechanical stress in the medial compartment, and the MCT inclination was correlated more strongly with the medial CBT of the proximal tibia than with the FTA.

MCT inclination has attracted attention as a parameter of knee OA. Matsumoto et al. [23] reported that the tibial plateau inclination was 86° in healthy and early osteoarthritic knees and 84° in advanced osteoarthritic knees, with a steeper inclination. It is assumed that continuous mechanical stress on the medial compartment due to physiological varus alignment gradually causes inclination of the proximal tibial articular surface, an increase in medial CBT, and progression of varus alignment, leading to the development and progression of varus knee osteoarthritis. In a 21-year longitudinal epidemiological survey, Higano et al. [1] noted that the tibial plateau angle and increased medial CBT of the proximal tibia were risk factors for the development of knee OA, but FTA was not a risk factor, indicating that changes in the MCT inclination and medial CBT of the proximal tibia occur before the onset of knee OA and earlier than the whole lower extremity alignment deformity. As shown in a previous study [1], the medial CBT of the proximal tibia and MCT inclination are probable predictors of knee OA onset and progression. Because the CBT is difficult to measure accurately on routine radiographs, MCT inclination may be a more practical parameter in the clinical setting.

This study has several limitations. First, the accuracy of this method depends on osteoporosis and CT scanning conditions such as pixel and voxel sizes. Treece et al. [2] demonstrated a high degree of accuracy by using a lower CBT of the femoral neck [2, 15]. The use of a greater CBT of the tibial diaphysis than that of the femoral neck in this study can be assumed to be reliable for measurement. Additionally, the size ranges of the pixels and voxels in this study were relatively narrow, limiting the effect of CT scanning conditions on the calculations of CBT and the reconstruction of 3D models. Second, the torsional deformity of the bone was not considered. When measuring the CBT, the region is divided based on the tibial coordinate system reconstructed by several proximal tibial landmarks, which may cause distal errors in knee OA due to torsional deformity. Finally, osteoporosis was not assessed using dual-energy X-ray absorptiometry, making it impossible to accurately evaluate the association between the CBT and osteoporosis. Further assessment should be conducted in the future.

In terms of clinical relevance, accurate verification using 3D-CBT, whole lower extremity alignment, and MCT inclination will lead to a better understanding of the pathological mechanism of knee OA.

## Conclusions

This study demonstrated that the medial CBT of the proximal tibia in osteoarthritic knees was thicker than that in healthy knees and correlated with FTA and MCT inclination, especially strongly with MCT inclination. The medial CBT of the proximal tibia and MCT inclination are probable predictors of knee OA onset and progression.

## Data Availability

No datasets were generated or analysed during the current study.

## Abbreviations

CBT: Cortical bone thickness
FTA: Femorotibial angle
MCT: Medial compartment of the proximal tibia
OA: Osteoarthritis
2D: Two-dimensional
3D: Three-dimensional
BMI: Body mass index
K-L: Kellgren-Lawrence
SD: Standard deviation
M/L: Medial/lateral
A/P: Anterior/posterior
